# Targeting Autophagy as a Strategy for Developing New Vaccines and Host-Directed Therapeutics Against Mycobacteria

**DOI:** 10.3389/fmicb.2020.614313

**Published:** 2021-01-14

**Authors:** Emily J. Strong, Sunhee Lee

**Affiliations:** Department of Microbiology and Immunology, University of Texas Medical Branch, Galveston, TX, United States

**Keywords:** autophagy, mycobacteria, host-directed therapies, non-tuberculous mycobacteria, host–microbe interactions, vaccines

## Abstract

Mycobacterial disease is an immense burden worldwide. This disease group includes tuberculosis, leprosy (Hansen’s disease), Buruli Ulcer, and non-tuberculous mycobacterial (NTM) disease. The burden of NTM disease, both pulmonary and ulcerative, is drastically escalating globally, especially in developed countries such as America and Australia. Mycobacteria’s ability to inhibit or evade the host immune system has contributed significantly to its continued prevalence. Pre-clinical studies have highlighted promising candidates that enhance endogenous pathways and/or limit destructive host responses. Autophagy is a cell-autonomous host defense mechanism by which intracytoplasmic cargos can be delivered and then destroyed in lysosomes. Previous studies have reported that autophagy-activating agents, small molecules, and autophagy-activating vaccines may be beneficial in restricting intracellular mycobacterial infection, even with multidrug-resistant strains. This review will examine how mycobacteria evade autophagy and discusses how autophagy could be exploited to design novel TB treatment strategies, such as host-directed therapeutics and vaccines, against *Mycobacterium tuberculosis* and NTMs.

## Introduction

While TB is a disease of significant global burden, the burden of non-tuberculosis mycobacteria (NTM) disease is higher than TB in many developed countries such as the United States and Australia (Prevots et al., 2010). NTMs are mycobacteria other than *Mycobacterium tuberculosis* (*Mtb*) and *Mycobacterium leprae* (the cause of leprosy/Hansen’s Disease). Globally, the burden of NTM continues to increase substantially. Like many pathogenic diseases, drug-resistance has become a severe public health concern for mycobacterial infection. In 2018, there were approximately 500,000 new rifampicin-resistant TB cases, most of which also comprised multiple drug-resistant infections (World Health Organisation, 2020). In contrast, the NTM species display significant heterogeneity in their susceptibility to standard anti-TB drugs and thus the treatment for NTM diseases usually involves the use of macrolides and injectable aminoglycosides. Although well-established international guidelines are available, treatment of NTM disease is mostly empirical and not entirely successful. In general, the treatment duration is much longer for NTM diseases, compared to TB. Taken together, the considerable global burden of mycobacterial disease requires much needed further research and the development of new treatment and prevention strategies.

The development of TB disease occurs in only 10% of individuals exposed to the pathogen, which infers that competent host defense mechanisms exist to control the infection. In the last decade, autophagy has surfaced as an essential host immune defense mechanism against intracellular *Mtb* infection. Autophagy is a complex, essential, conserved cellular process allowing for the degradation of intracellular components, including proteins, organelles, and foreign bodies. Autophagy targeting by host-directed therapies to enhance treatment options against pathogenic viruses and bacteria has recently become a popular research topic. Similarly, autophagy has been proven not only as an effective antimicrobial mechanism for the clearance of *Mtb* and NTMs, but as a process preventing excessive inflammation to avoid adverse effects of infection on the host. Still, increasing evidence shows that in order to augment its intracellular survival, mycobacteria has evolved multiple strategies to prevent the optimal operation of host autophagic machinery.

This review will focus on autophagy during mycobacterial infection. However, it is worth noting that many intracellular pathogens are known to modulate autophagy to promote their survival. For example, *Legionella pneumophila* secrets bacterial effector that irreversibly inactivates Atg8 proteins unable to be reconjugated by the Atg7-Atg3 (Choy et al., 2012). Many other intracellular bacteria like *Shigella*, *Salmonella*, and *Mycobacteria* also secrete bacterial effectors that inhibit autophagy (Ogawa et al., 2005; D’Cruze et al., 2011; Popa et al., 2016; Saini et al., 2016; Jiao and Sun, 2019; Strong et al., 2020). A deeper understanding of the mechanisms by which these bacteria cause disease should foster better treatment options. Ongoing analysis is even more critical, given the rising infection rates of NTMs and rapidly growing mycobacteria (RGM), increased prevalence of drug-resistant TB, and TB/Diabetes and TB/HIV comorbidity. This review will cover the current understanding of the molecular mechanisms by which mycobacteria can modulate autophagy. Additionally, it will discuss the potential for these insights to be utilized and harnessed to develop host-directed therapies as treatment options against mycobacterial diseases.

## Autophagy Pathway as a General Antimicrobial Defense

**FIGURE 1:**
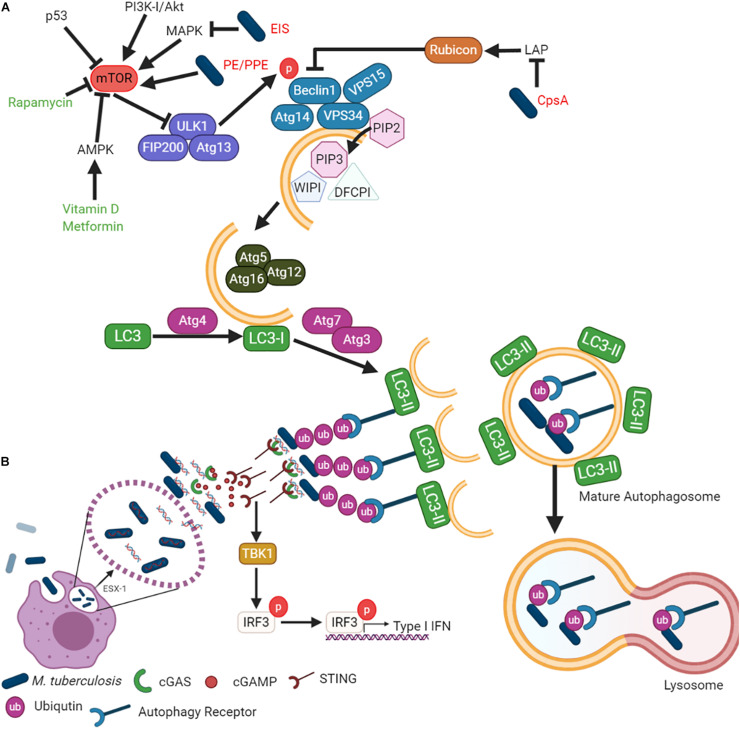
*Mycobacterium tuberculosis* inhibits autophagy to enhance survival in host cells. **(A)** While autophagy can be explained in terms of its role in cell survival, the mechanism by which it is regulated is complicated and elaborate. The different steps of the autophagic pathway during mycobacterial infection are shown. **(B)** *Mtb*-autophagy interaction in macrophages. Following phagocytosis, *Mtb* resides in phagosome and blocks phagosome maturation. *Mtb* secretes Esx-1, promoting phagosome damages that trigger ubiquitination, recruitment of autophagic adaptors and mycobacterial capture via STING. The detailed molecular mechanisms of each steps and mycobacterial factors are discussed in the text.

Macroautophagy is the most widely studied form of autophagy and is an evolutionarily conserved pathway controlling quality and quantity of eukaryotic organelles and the cytoplasmic biomass (Svenning and Johansen, 2013). Macroautophagy involves the formation of a double membrane phagosome, which fuses with a lysosome (Parzych and Klionsky, 2013). It is a constitutive cellular process that is induced under stress conditions such as nutrient starvation, which degrades cytoplasmic material into metabolites and degrades cytoplasmic foreign bodies (Svenning and Johansen, 2013). Macroautophagy can be selective, as it recognizes specific marked components by various receptor proteins such as p62 (SQSTM1) (Svenning and Johansen, 2013). The degradation of pathogens is called Xenophagy, whereby bacteria are engulfed by autophagosomes and degraded after fusion with lysosomes to form autolysosomes. This review will focus on Xenophagy, which will hereafter be referred to as “autophagy.” The autophagy pathway is illustrated in Figure 1A, showing the minimal core components relevant for the discussion in this review.

The formation of the autophagosome and the fusion to a lysosome is broken down into five main steps (Figure 1A): (i) initiation, (ii) elongation, (iii) maturation, (iv) fusion, and (v) degradation. Autophagy initiation is regulated by the master regulator, the mammalian/mechanistic target of Rapamycin (mTOR). It is a negative regulator of autophagy, meaning its dephosphorylation is responsible for autophagy induction. Dephosphorylation of mTOR results in the translocation of the Unc-51 like autophagy activating kinase (Ulk1/2)-Autophagy related (Atg)13-FAK family-interacting protein (FIP200)-Atg101 complex to the endoplasmic reticulum (Itakura and Mizushima, 2010; Mizushima, 2010). The Class III phosphatidylinositol 3 kinase (PI3K) activates the VPS34-Beclin1-VPS15-Atg14 complex. The PI3K complex induces phosphatidylinositol-3-phosphate [PtdIns(3)P], which then recruits double FYVE-containing protein 1 (DFCP1) and WD-repeat domain phosphoinositide-interacting (WIPI) family proteins to initiate the omegasome formation (Axe et al., 2008; Matsunaga et al., 2009; Itakura and Mizushima, 2010; Polson et al., 2010). Elongation of the omegasome into the autophagosome is conducted by the Atg7–Atg10 complex and then conjugated to Atg12-Atg5-Atg16L on the omegasome membrane (Fujita et al., 2008). Atg4 cleaves LC3 into LC3-I, while the Atg7–Atg3 complex lipidates LC3-I into LC3-II by conjugating phosphatidyl-ethanolamine (PE). The completed autophagosome fuses with the lysosome to degrade the autophagosome cargo for subsequent metabolite recycling or antigen presentation (Knodler and Celli, 2011; Levine et al., 2011; Münz, 2016; Saini et al., 2016; Yu et al., 2018).

## Autophagy and Tuberculosis

### Autophagy Induction by *M. tuberculosis*

The global prevalence of mycobacterial diseases of all types has increased considerably. The most significant mycobacterial disease is tuberculosis (TB). In 2018, 1.4 million deaths were attributed to *Mtb* infection (World Health Organisation, 2020), meaning TB is one of the top 10 causes of death and the leading cause of worldwide death from a single infectious agent. One-quarter of the world’s population is infected with *Mtb.* Approximately 5 to 10% of infections progress to the active disease at some point in their host lives. *Mtb* is a successful pathogen due to its capacity to evade the host immune systems and utilize phagocytes as a replication niche. The bacteria can significantly inhibit the phagolysosome’s acidification and limit phagosome maturation, thereby forcing the infected cell to undergo programmed cell death (Russell et al., 2002). Several of the seminal observations regarding the antimicrobial role of autophagy have been made using *Mtb* (Gutierrez et al., 2004; Castillo et al., 2012) and such observations were followed by a gradual increase in studies on autophagy as a cell-autonomous, pharmacologically, physiologically and immunologically inducible anti-mycobacterial process (Gutierrez et al., 2004; Seto et al., 2012; Watson et al., 2012; Sakowski et al., 2015). These studies revealed the colocalization of *Mtb* with autophagosomes and increased bacterial clearance during autophagy induction (Gutierrez et al., 2004; Seto et al., 2012; Watson et al., 2012; Sakowski et al., 2015). Most of these models have been explored in the context of Atg5 and its effect on autophagy. Examination of other important autophagy markers such as Ulk1 and Atg4b *in vivo* has uncovered that Atg5 may play a role independent of canonical autophagy in *Mtb* control (Kimmey et al., 2015).

Infection with mycobacteria induces significant levels of pro-inflammatory cytokines, known to be inducers of autophagy. However, little colocalization of *Mtb* and autophagosomes has been observed without non-mycobacterial stimulation of autophagy. An example of immunological induction of mycobactericidal autophagy includes stimulation of infected macrophages with Th1 cytokines such as IFN-γ and TNF-α in a process that can be antagonized by Th2 cytokines including IL-4 and IL-13 (Harris et al., 2007; Ghadimi et al., 2010). Physiological induction of autophagy by IFN-γ generated significant autophagosome formation in mycobacteria-infected macrophages and dendritic cells. Although *Mtb* infection causes a robust IFN-γ response, the autophagy induction by virulent *Mtb* is limited, probably because mycobacteria inhibit IFN-γ mediated autophagy induction (Zullo and Lee, 2012a; Zullo et al., 2014). IFN-γ also plays a critical role in the nitric oxide (NO) response to *Mtb* infection. The deletion of IFN-γ significantly impedes NO production and leads to uncontrolled replication of bacilli *in vivo* (Cooper et al., 1993; Flynn et al., 1993). The inhibition of NO has previously been shown to induce autophagy substrates’ clearance, highlighting the complex role of cytokine signaling in autophagy pathways (Sarkar et al., 2011). Virulent *Mtb* infection induces TNF-α, which is inactivated by the increased release of TNFR2 and results in inhibition of apoptosis (Balcewicz-Sablinska et al., 1998; Keane et al., 2000). This observation underscores the potential of virulent *Mtb* to inhibit autophagy by modulating cytokines’ bioreactivity known to induce autophagy.

### Manipulation of Autophagy Pathways by Mycobacterial Factors

Several bacterial effector proteins are known to modulate autophagy. Many of these effectors are secreted through the type I to type VII and type IX secretion systems (Jiao and Sun, 2019). Mycobacteria has numerous Type VII secretion systems (Esx1 – Esx5). *Mtb* ESX-1 is responsible for the puncture of the phagosome, allowing for mycobacterial escape (Conrad et al., 2017) (Figure 1B). *Mtb* cytosolic DNA is recognized by the cytosolic DNA sensor, cyclic GMP-AMP synthase (cGAS), resulting in the release of cyclic guanosine monophosphate (cGAMP). cGAMP is recognized by the stimulator of interferon genes (STING), leading to type I IFN release and the recruitment of autophagy receptors p62, NDP52, and optineurin (Watson et al., 2012, 2015). These receptors are recruited to the ubiquitinated pathogen, thereby allowing for specific targeting by the autophagosome. The receptors contain an LC3 interaction region (LIR) to bind the LC3 autophagy protein (Thurston et al., 2009; Zheng et al., 2009; Wild et al., 2011).

EspB is a part of the Esx1 secretory apparatus responsible for the secretion of early secretory antigenic target-6 (ESAT-6). Treatment of macrophages with EspB protein demonstrates downregulation of the IFN-γ receptor IFN-γR1, resulting in the inhibition of STAT-1 activation even in the presence of IFN-γ (Huang and Bao, 2016). EspB and ESAT-6 are not the only *Mtb* proteins linked to the inhibition of autophagy. The “enhanced intracellular survival” (*eis*) gene of *Mtb* can confer enhanced survival of *Mycobacterium smegmatis* in macrophages. However, it is not required for the persistence of *Mtb* in these cells (Wei et al., 2000; Shin et al., 2010). During *Mtb* infection, *eis* significantly inhibits the activation of JNK, which prevents the induction of non-canonical autophagy through Atg7. JNK activation also induced reactive oxygen species (ROS) generation and significantly increased type 2 macrophage cell death by *Mtb eis* deletion mutant (Shin et al., 2010). Eis was also found to substantially inhibit the production of TNF-α, IL-4, and IL-6, while simultaneously stimulating INF-γ and IL-10 secretion (Lella and Sharma, 2007; Samuel et al., 2007; Shin et al., 2010).

*Mtb* inhibits autophagy to protect against bacterial clearance and host cell death, which also impedes antigen presentation. The *Mtb* PE_PGRS47 protein inhibits autophagy and limits MHC class II antigen presentation (Saini et al., 2016). Several other *Mtb* PE/PPE proteins are also known to inhibit autophagy. For example, *Mtb* PE_PGRS41 (Deng et al., 2017), *Mycobacterium marinum* MMAR_0242 (Singh et al., 2016), and *Mtb* PE_PGRS29 (Chai et al., 2019) interact with autophagy machinery. *Mtb* also secrets a probable ligase (CpsA) to inhibit the non-canonical autophagy pathway designated as LC3-associated phagocytosis (LAP) and NADPH oxidase (Köster et al., 2017) (Figure 1A). In contrast to canonical autophagosomes, LAP does not result in double-membrane structures and instead promotes rapid phagosome maturation (Fazeli and Wehman, 2017). This cellular process limits the phagocytosed pathogen’s ability to replicate by expediting phagosome maturation while regulating the IFN pathway and antigen presentation.

Additionally, studies have found that virulent *Mtb*, but not avirulent *Mtb*, can inhibit autophagy flux in macrophages and dendritic cells in an ESAT-6 and PhoP dependent manner (Chandra et al., 2015). Autophagy flux is an important cellular mechanism that degrades autophagosome cargo, which allows nutrient recycling or antigen presentation. Increased autophagic flux was found to improve bacterial clearance from macrophages and dendritic cells (Romagnoli et al., 2012; Chandra et al., 2015). The maturation of *Mtb*-containing autophagosomes into autolysosomes was inhibited by blocking recruitment of the late endosome marker Rab7 (Chandra et al., 2015). Inhibition of Rab5 conversion to Rab7 in endosomes is a well-established method in which mycobacteria inhibit lysosomal fusion (Via et al., 1997; Rink et al., 2005).

*Mtb* inhibits canonical and non-canonical autophagy by several means and it is apparent that the role of infection-induced autophagy is complicated. While overcoming autophagy inhibition by *Mtb* could lead to better treatment options, further consideration should be given to evidence suggesting that *Mtb* can inhibit autolysosome formation (Chandra et al., 2015). Additionally, there may be value in examining host-directed therapies targeting mTOR-independent autophagy pathways (Schiebler et al., 2015), since *Mtb* infection markedly activates mTOR. Exploring alternative autophagy-inducing pathways may lead to more efficacious drugs and may prove more useful in patients presenting with co-disease such as TB/Diabetes.

## Autophagy and Non-Tuberculosis Mycobacteria

### NTM Disease

NTM is the broad term for diseases caused by over 170 mycobacteria. The most commonly isolated specie is the *Mycobacterium avium* Complex (MAC), accounting for 71.1% of Australian cases and 31.3% of NTM cases in South America. *M. avium* is the most common species isolated in Europe, Asia, South America, and North America. At the same time, *M. intracellulare* is prevalent in South Africa and Australia (Johnson and Odell, 2014; Griffith, 2019; Gopalaswamy et al., 2020). *M. avium*, MAC, and *M. intracellulare* most commonly present as a pulmonary disease similar to TB. In 2013, a nearly six-fold increase in NTM cases were reported in America compared to the 1980s (Donohue and Wymer, 2016), with similar trends in the United Kingdom, Denmark, and Germany (Ratnatunga et al., 2020). Some studies have attributed this rise of NTM infections to a vaccination policy change from a blanket BCG vaccination to a limited vaccination only for specific groups (SAGE Working Group of BCG Vaccines and WHO Secretariat, 2017; Kontturi et al., 2018).

Other common NTMs include *Mycobacterium fortuitum*, *Mycobacterium kansasii*, *Mycobacterium abscessus*, *M. marinum*, and *Mycobacterium ulcerans*. NTMs are opportunistic environmental pathogens that are typically found in soil and water. Although NTM disease presentation is most commonly pulmonary, observation of lymphatic, skin/soft tissue and disseminated disease have been reported (Griffith et al., 2007; Bodle et al., 2008; Tortoli, 2009). Many species of mycobacteria can also cause ulcerative disease. Four main presentations of the mycobacterial ulcerative disease have been designated: (i) cutaneous *Mtb* infection, (ii) leprosy (the second most common mycobacterial disease; caused by *M. leprae* or *Mycobacterium lepromatosis*), (iii) Buruli Ulcer, the third most common mycobacterial disease (caused by *M. ulcerans*), and (iv) opportunistic infections caused by other non-tuberculosis mycobacteria such as *M. marinum*. Franco-Paredes et al. (2018) eloquently summarized the disease presentation of cutaneous mycobacterial infections in their 2018 review.

Besides *Mtb*, *M. leprae* and *M. ulcerans* account for the next highest mycobacterial disease burdens. Buruli Ulcer, caused by *M. ulcerans*, primarily occurs in the West and Central Africa, Asia, South America, the western Pacific, and Australasia (Simpson et al., 2019). Unlike the well-studied *Mtb* and *M. leprae*, the mode of transmission of *M. ulcerans* remains unknown (Röltgen and Pluschke, 2015). As with other mycobacteria, treatment of Buruli Ulcer is costly and takes a long time. Traditional antimycobacterial antibiotics are used for treatment, including rifampicin, streptomycin, clarithromycin, and moxifloxacin. However, wound interventions, such as lymphedema management and surgery, are commonly used to speed up healing (Yotsu et al., 2018; Converse et al., 2019; World Health Organisation, 2019). Though leprosy transmission remains on the decline with less than 200,000 cases in 2017 (World Health Organization, 2016), improved treatment options are a vital resource for continued disease decline (Fischer, 2017; Maymone et al., 2020; Scollard, 2020). Leprosy broadly presents two different clinical manifestations; paucibacillary tuberculoid, which is characterized by negative smears for acid-fast bacilli, and multibacillary lepromatous, which is characterized by positive smears for acid-fast bacilli (Nath, 2016).

Mycobacteria’s unique cell wall and some species’ ability to form biofilms, spread by aerosolization, slow growth, and intrinsic antibiotic resistance, also contribute to their ability to survive in unique and low nutrient environments (De Groote and Huitt, 2006). Their lipid-rich cell wall influences the bacteria’s ability to modulate autophagy (Zullo and Lee, 2012a). The ability to form biofilms and survive in low nutrient environments indicates that these bacteria can form unique replication niches within the hosts’ cells that traditional mycobacterial drugs cannot penetrate to be effective (Islam et al., 2012).

### NTM and Autophagy

The induction of autophagy by mycobacteria is species-dependent. Although all mycobacteria elicit strong mTOR activation, most non-pathogenic mycobacteria simultaneously induce significant autophagy, unlike their pathogenic relatives (Zullo and Lee, 2012a). *M. smegmatis* is often utilized as a model organism to study pathogenic mycobacteria due to its short culture time and BSL2 classification (Deng et al., 2017). While a low concentration of mTOR-inhibiting drugs like Rapamycin and Torin are able to inhibit mTOR activation and induce autophagy during mycobacterial infection (Zullo et al., 2014), clearance of *M. smegmatis* requires up to 10 times higher quantity of those drugs than needed to inhibit mTOR activation. Interestingly, this killing was observed to be independent of LC3B or Atg5, indicating a non-canonical autophagy pathway is involved in the clearance of *M. smegmatis* from macrophages (Zullo et al., 2014). This interesting observation suggests that targeting a non-canonical autophagy pathway for mycobacterial treatment may be useful. It has previously been shown that treatment of *Mtb* infected macrophages with potent autophagy inducers such as *M. smegmatis* can clear bacteria (Singh et al., 2017).

The role of autophagy during NTM has not been studied extensively. However, evidence exists that genetic variants in the autophagy-related genes, nucleotide-binding oligomerization domain-containing 2 (NOD2), E3 ubiquitin-protein ligase parkin (PARK2), IRGM, and autophagy-related proteins 16-1 (ATG16L1), are associated with susceptibility to mycobacterial disease (Yang et al., 2014; Capela et al., 2016; Uaska Sartori Priscila et al., 2020). A single nucleotide polymorphism (SNP) in PARK2 correlates significantly with increased susceptibility to *M. ulcerans* infection, while an SNP in NOD2 is associated with increased disease progression. Conversely, an SNP in ATG16L1 protects against severe disease during *M. ulcerans* infection (Capela et al., 2016; Manry et al., 2020). Although not directly associated with autophagy, other SNPs in iNOS and IFN-γ have been associated with increased susceptibility to Buruli Ulcer, leprosy, and TB (Bibert et al., 2017).

The major virulence factor of *M. ulcerans* is mycolactone, a cytotoxic, immunosuppressive polyketide-derived macrolide. Mycolactone alone induces autophagy, although it impairs autophagy flux (Gama et al., 2014). The induction of autophagy is further evidenced by mycolactone’s ability to inhibit mTOR, thereby resulting in the upregulation of apoptosis activating protein, Bim (Bieri et al., 2017). This pathway signals through the inactivation of Akt by an alternative mTOR pathway. As such, activation of mTOR could lead to inhibition of Bim and, subsequently, apoptosis, resulting in control of bacterial infection.

Two variants of *M. abscessus* and *M. fortuitum* are frequently observed: rough (R) and smooth (S) (Byrd and Lyons, 1999; Catherinot et al., 2007; Lee et al., 2016). It is widely accepted that the R variant is hypervirulent compared to its S counterpart. It is known that the loss of glycopeptidolipid (GPL) is the cause of the S-variant of *M. abscessus* in several animal models (Byrd and Lyons, 1999; Catherinot et al., 2007). A highly virulent clinical isolate of *M. abscessus*-R significantly inhibited autophagic flux than the S variant of *M. abscessus*. The R variant’s intracellular survival is enhanced considerably by blocking the autophagosome-lysosome fusion in macrophages compared to the S variant (Kim et al., 2017c). These immunological effects of NTMs have been mostly studied from the perspective of respiratory illness and genome comparison studies focusing on traditional virulence factors for related opportunistic pathogens (N’Goma et al., 2015) indicates we have only a minimal understanding of their impact during ulcerative infection.

Whereas *M. abscessus* S utilizes phosphatidyl-*myoinositol* to mask TLR2 activation, *M. fortuitum* R does not induce the anti-inflammatory molecule TNFAIP3 (Lee et al., 2016). TNFAIP3 is an anti-apoptotic molecule that inhibits NF-κB and TNF-induced cell death (Lee et al., 2000). TNF- α and the TLR2 signaling pathway appear to play an essential role in *M. fortuitum* infection. Some lipids of RGMs have differential terminal modifications compared to those from pathogenic slow-growing mycobacteria. Specifically, lipoarabinomannan (LAM) in RGM is capped with phosphomyo-inositol (PI) caps compared to mannose (Man) caps in pathogenic mycobacteria. Purified PI-LAM induces significantly more apoptosis than purified Man-LAM in a TLR2 dependent manner (Bohsali et al., 2010). Similarly, PILAM caused significant autophagy induction, unlike ManLAM, which did not induce autophagy (Shui et al., 2011; Singh et al., 2019). Although terminal modifications of LAM appear to play a role in the modulation of apoptosis, total lipid from both pathogenic and non-pathogenic mycobacteria can induce autophagy (Zullo et al., 2014; Kim et al., 2017c; Mishra et al., 2019). Interestingly, while total lipids from *M. abscessus*-R induce a significant autophagy level, live *M. marinum* induces autophagy and simultaneously inhibits autophagy flux, which leads to increased intracellular survival (Lerena and Colombo, 2011; Kim et al., 2017c; Oliveira et al., 2020; Pohl et al., 2020).

It has been known for many years that autophagy is an efficient mechanism to clear *M. leprae* from macrophages (Evans and Levy, 1972). However, it has been recently described that autophagy may be a major modulating factor in leprosy disease presentation. In patients presenting with multibacillary leprosy, there is significantly less autophagic control in macrophages taken from patient lesions than patients presenting with paucibacillary tuberculoid leprosy (Silva et al., 2017a). This supports previous studies which found that the autophagy inhibiting cytokine IL-10 is predominant in multibacillary leprosy compared to high levels of IL-26, IFN-γ, and TNF-α, autophagy inducing cytokines, found during paucibacillary tuberculoid leprosy (Yamamura, 1992; Sieling and Modlin, 1994; Nath, 2016; Dang et al., 2019). Multibacillary leprosy patients who developed type 1 reaction (T1R) episodes demonstrated dysregulation of autophagy genes and significantly increased expression of the mTOR complex leading to overexpression of the NLRP3-inflammasome-IL-1B pathway. These data demonstrate that leprosy treatment with pro-autophagic drugs may improve treatment outcomes by reducing reversal reaction risk (de Mattos Barbosa et al., 2018).

The establishment of uncontrolled mycobacterial infection in an extracellular bacterial milieu or biofilm presents significant complications for treatment (Greendyke and Byrd, 2008). Many mycobacterial species causing ulcerative diseases are widely considered to have significantly reduced sensitivity to antibiotics and a natural ability to acquire antibiotic resistance, making it very hard to treat and leading to high failure rates (Moore and Frerichs, 1953; Jarlier and Nikaido, 1990; Sanguinetti et al., 2001; Nessar et al., 2012). Utilizing host-directed therapies, such as those inducing autophagy, to inhibit bacterial release from the cell and form biofilms or bacterial milieus may enhance the efficacy of currently available antibiotics.

## Harnessing Autophagy to Fight Mycobacteria

### Targeting Autophagy to Treat a Mycobacterial Infection

Rapamycin has been the most frequently used autophagy-inducing drug for host-directed therapies. While Rapamycin appears to improve pathology during *Mtb* infection, there is evidence that it is directly antimycobacterial *in vitro* at the high concentration used for the reported studies. Rapamycin does not seem to have a direct effect on *M. smegmatis* or BCG for short periods. Still, it was found to significantly inhibit BCG, *M. kansasii, M. avium*, and multiple virulent *Mtb* strains over 7–8 days incubation (Greenstein et al., 2008; Zullo et al., 2014). This direct antimycobacterial activity is somewhat unsurprising as Rapamycin was initially discovered as a novel antifungal antibiotic (Singh et al., 1979). Rapamycin is not the only drug evaluated as a host-directed therapy for the treatment of tuberculosis. Some medications, such as azithromycin and metformin, have been found to decrease mycobacterial infections in patients with cystic fibrosis and diabetes due to their ability to increase the autophagic clearance of bacteria (Renna et al., 2011; Tseng, 2018). Table 1 summarizes the drugs and compounds that have been tested for their ability to induce autophagy and treat mycobacterial diseases.

**TABLE 1:** Summary of current experimental treatments inducing autophagy during *M. tuberculosis* infection.

Drug	Model	Mode	References
Small Molecule Enhancers of Rapamycin	*M. bovis* BCG infection of primary human macrophages	Induce autophagy independently of mTOR	Floto et al., 2007
Rifampicin	*Mtb* infection of human differentiated monocytes	Increased autolysosome formation, directly antimycobacterial	Genestet et al., 2018
Linezolid	*Mtb* infection of human differentiated monocytes	Increased autophagosomes production, directly antimycobacterial	Genestet et al., 2018
Bedaquiline	*Mtb* infection of human differentiated monocytes	Increased autophagosomes production, directly antimycobacterial	Genestet et al., 2018
Nitazoxanide	*Mtb* infection of human differentiated monocytes. *M. leprae* infection of mice	Increased autophagy by inhibition of NADPH quinone oxidoreductase 1 leading to mTOR inhibition by TSC2	Lam et al., 2012; Bailey et al., 2017
Baicalin	*Mtb* infection of mouse macrophages	Induce autophagy via the PI3K/Akt/mTOR pathway, inhibit NLRP3 inflammasome activation via the PI3K/Akt/NF-κB, reduction of proinflammatory cytokines	Lin et al., 2013; Zhang et al., 2017
Vitamin D	*Mtb*/HIV co-infection model of primary human macrophages	Cathelicidin dependent induction of autophagy	Liu et al., 2007; Martineau et al., 2007; Yuk et al., 2009; Jo, 2010; Campbell and Spector, 2012b
4-phenylbutyrate	*Mtb* infection of human monocytes	Induction of LL-37 promoting autophagy via P2RX7 receptor, increasing free Ca^2+^ and activation of AMPK and PtdIns3K pathway.	Rekha et al., 2015
Gefitinib	*Mtb* infection of murine bone marrow-derived macrophages	STAT3 dependent cytokine responses, increasing lysosomal trafficking	Stanley et al., 2014; Sogi et al., 2017
Carbamazepine	*Mtb* infection of human-derived macrophages or murine alveolar macrophages. *M. marinum* zebrafish model of infection. MDR *Mtb* infection of C57BL/6 mice	Induce autophagy by blocking myoinositol uptake, decreasing phosphatidylinositol, and activating AMP kinase in an mTOR independent manner.	Schiebler et al., 2015; Juárez et al., 2016
Valproic acid	*Mtb* infection of human-derived macrophages or murine alveolar macrophages	Increases colocalization of LC3 with *Mtb*	Schiebler et al., 2015; Juárez et al., 2016
Loperamide	*Mtb* infection of human-derived macrophages or murine alveolar macrophages	Increases colocalization of LC3 with *Mtb* and reduces TNF-α production	Juárez et al., 2016
Simvastatin	*Mtb* infection of C57BL/6 mice	Reduction of membrane cholesterol levels promotes phagosomal maturation and autophagy	Parihar et al., 2013
Metformin	*Mtb* infection of C57BL/6 mice	Induction of mitochondrial reactive oxygen species, AMPK activation, and autophagy induction	Singhal et al., 2014; Restrepo, 2016
Trehalose	*Mtb*/*M. avium*/*M. fortuitum* infection of human differentiated monocytes	Increase autophagy flux through activation of ptdIns3P by activation of PIKFYVE	Sarkar et al., 2007; Sharma et al., 2020
Mycobacterial PILAM	*Mtb* infection of murine macrophages	Induction of autophagy and pro-inflammatory cytokines, enhanced colocalization of *Mtb* with phagolysosomes	Shui et al., 2011; Singh et al., 2019
Nordi-hydroguaiaretic acid	Avirulent *Mtb* infection of human differentiated monocytes	Directly antimycobacterial, induce autophagosome formation and colocalization with *Mtb*	Guzmán-Beltrán et al., 2016
Lactoferricin peptides	*M. avium* infection of murine bone marrow macrophages	Increased autophagosome formation	Silva et al., 2017b

Of importance, the use of Rapamycin to treat infectious diseases is not practical due to its immunosuppressant actions. Gupta et al. (2014) have attempted to address this issue with the administration of Rapamycin by microparticles directly to the airway. Highlighting the delicate balance needed for host-directed therapies, the study found that the induction of autophagy in the lung macrophages was inverse to the dosing interval. *In vitro* and *in vivo* rapamycin microparticles induce autophagolysosomal formation in macrophages infected with *Mtb* in an mTOR-dependent manner (Gupta et al., 2014; Gupta et al., 2016). Rapamycin alone significantly improved pathology during *Mtb* infection in a mouse model but did not clear bacteria. Co-administration of isoniazid and rifabutin with Rapamycin microparticles considerably improved bacterial clearance (Gupta et al., 2014, 2016). As the administration of microparticles with autophagy-inducing drugs may improve traditional antimycobacterial chemotherapies, an alternative strategy is utilizing microparticles that directly induce autophagy. Poly (lactic-co-glycolic acid) microparticles were found to be antimycobacterial in human macrophages. For example, NFκB activity was increased during microparticle treatment, and antimycobacterial effects were reversed by autophagy inhibitors (Lawlor et al., 2016).

An antiprotozoal drug, nitazoxanide, has been extensively tested to treat *Mtb* and NTMs alone and in conjunction with traditional antimycobacterial medicines. These conventional antibiotics were not found to inhibit autophagosome formation stimulated by nitazoxanide (Lam et al., 2012). Nitazoxanide has been previously explored as an autophagy agonist for treating multiple disease states such as Alzheimer’s and cancer (Di Santo and Ehrisman, 2014; Li et al., 2020). It has also been examined as a potential treatment option against several mycobacteria including *M. leprae* (Bailey et al., 2017) and MAC (Rossignol, 1999). Nitazoxanide is metabolized into hydroxylamine by mycobacterial nitroreductase NfnB (Buchieri et al., 2017). Interestingly, nitazoxanide can kill replicating and non-replicating mycobacteria, emphasizing its potential role in combating latent mycobacterial infection (de Carvalho et al., 2009; Iacobino et al., 2019). Like Rapamycin and nitazoxanide, metformin also increases bacterial clearance during traditional anti-mycobacterial treatment, while inducing autophagy (Singhal et al., 2014; Lachmandas et al., 2019).

Two of the most promising experimental host-directed therapies against *M. tuberculosis* are Vitamin D3 and Metformin (Naicker et al., 2020). Metformin was shown to increase mitochondrial reactive oxygen species production, acting through AMPK, leading to control of drug-resistant *Mtb* and facilitation of phagolysosome fusion (Singhal et al., 2014; Yew et al., 2020). There also appears to be a correlation between metformin treatment for diabetes mellitus type II and delayed smear and culture conversion and reduced unfavorable outcomes (Singhal et al., 2014; Degner et al., 2017; Marupuru et al., 2017; Lee et al., 2018; Padmapriyadarsini et al., 2019). While metformin shows promise in preventing TB in type II diabetes patients, Vitamin D supplementation showed no improvement in TB treatment outcomes in patients with vitamin D sufficiency during drug sensitive *Mtb* infection. However, vitamin D deficiency is associated with an increased risk of *Mtb* infection (Ustianowski et al., 2005; Chun et al., 2011). Vitamin D supplementation did reduce the time to sputum culture conversion in patients with Taql vitamin D receptor gene polymorphism, indicating that Vitamin D does play an important role in TB treatment outcomes. Vitamin D supplementation also improved the MDR-TB sputum culture conversion rate (Zhang et al., 2019). *In vitro* treatment with vitamin D during HIV and *Mtb* co-infection or *Mtb* infection alone concluded that autophagy induction was responsible for the better control of both HIV and *Mtb* in macrophages (Yuk et al., 2009; Fabri et al., 2011; Campbell and Spector, 2012a).

Many host pathways may constitute viable targets for host-directed therapies (HDTs). Apoptosis and autophagy have been the most explored HDT targets of *Mtb* and NTMs. Even though apoptosis may be a possible target, there is mounting evidence that NTMs can escape apoptotic bodies to ensure survival and disease progression (Early et al., 2011; Bento et al., 2020). Autophagy presents an exciting target as the induction of autophagy promotes bacterial clearance and antigen presentation (Castillo et al., 2012; Saini et al., 2016). The current recommended treatment for NTM infection is clarithromycin or azithromycin, ethambutol, and rifamycin (Griffith, 2018; Griffith, 2019; Daley et al., 2020; Gopalaswamy et al., 2020). Azithromycin was shown to inhibit autophagosome maturation resulting in an increased risk of *M. abscessus* infection (Renna et al., 2011; Torfs et al., 2019). It has also been found that many traditional anti-mycobacterials, though directly antimycobacterial, also have off-target effects that promote autophagy (Kim et al., 2012; Zullo and Lee, 2012b). Unfortunately, the development of new drugs targeting particular host pathways is often slow and expensive. One potential strategy to expedite this drug discovery is studying and assessing previous medications known to increase autophagy and their effect on mycobacteria (Williams et al., 2008; Sundaramurthy et al., 2013; Stanley et al., 2014; Kim et al., 2019). Potentially repurposed autophagy targeting host-directed therapies are summarized in Table 2. Many of these drugs are of interest because of the modulation of the host immune response. Accordingly, they should also be effective against a broad range of mycobacteria and other intracellular pathogens.

**TABLE 2:** Summary of repurposed autophagy targeting host-directed therapies against NTMs.

Drug	Model	Role in Autophagy	Reference
Azithromycin	*M. abscessus* infection of primary human macrophages and C57BL/6 mice	Blocks lysosomal acidification, impairing autophagosome degradation, directly antimycobacterial on susceptible strains	Renna et al., 2011
Carvacrol	Directly antimycobacterial against *M. abscessus*, *M. chelonae*, *M. fortuitum*, *M. mucogenicum*, *M. avium* sbsp paratuberculosis, and *M. smegmatis*	MEK inhibition of mTOR resulting in increased autophagy. Inhibition of autophagy during adipogenic differentiation.	Nowotarska et al., 2017; Potoènjak et al., 2018; Spalletta et al., 2018; Marini et al., 2019
Tetracycline	Directly antimycobacterial against *M. abscessus*, *M. chelonae*, and *M. fortuitum*	mTOR inhibition	Brüning et al., 2014; Kaushik et al., 2019; Shoen et al., 2019
Thioridazine	*In vitro* clearance of *M. avium* from Thp-1 macrophages, possible efflux pump inhibitor	Upregulation of AMPK activity leading to autophagy induction.	Rodrigues et al., 2008; Deshpande et al., 2016; Seervi et al., 2018; Chu et al., 2019
Mefloquine	Directly antimycobacterial against *M. avium* complex in culture. *In vivo* clearance in mice	Highly induced formation of autophagosomes in neuroblastoma cells	Bermudez et al., 1999, 2012; Shin et al., 2012
Clonidine/Verapamil/Minoxidil	Decreased disease progression in *in vitro*, fly, and zebrafish models of infection	Clearance of soluble huntingtin exon 1 during *M. leprae* infection by autophagy	Williams et al., 2008

As a better understanding of the role of infection-induced autophagy transpires, more targeted host-directed therapy approaches can be developed and exploited. With C_4_T_4_ (a TLR4 agonist), autophagy was induced in guinea pigs infected with *Mtb* in a CLEC4E-dependent manner through MYD88 and PrdIns3K activation, leading to reduced mycobacterial burden (Pahari et al., 2020). Along with targeting cell receptors to activate autophagy, there is increasing evidence that many microRNAs (miRNAs) can be targeted to activate autophagy during mycobacterial infection (Wang et al., 2013; Kim et al., 2015, 2017a,b; Kumar et al., 2016; Etna et al., 2018; Liu et al., 2018; Li et al., 2019). These miRNAs modulate autophagy through different upstream pathways of mTOR. *Mtb* infection induces miRNA-144, which targets the DNA damage regulated autophagy modulator 2 (DRAM2), resulting in autophagy inhibition through AMPK (Kim et al., 2017b). Similar to targeting CLEC4E through TLR4 agonists, TLR2 and MYD88 are required to induce miRNA-125a during *Mtb* infection. *Mtb* induces expression of miR-125a in macrophages, which results in the inhibition of autophagy by targeting UV radiation resistance-associated gene (UVRAG) in the AMPK dependent manner (Kim et al., 2015).

### Targeting Autophagy to Prevent Mycobacterial Infection

BCG is widely used as a vaccine against tuberculosis. BCG evades phagosome maturation, autophagy, MHC-II expression of antigen-presenting cells (APCs), and T-cell activation (Deretic et al., 1997; Singh et al., 2006; Münz, 2016; Saini et al., 2016; Khan et al., 2019). Clinical isolates of *Mtb* that could not inhibit autophagy showed increased TB disease outcomes and the extent of disease (Li et al., 2016), strongly indicating that autophagy is a crucial host pathway for the control of TB. The ability to utilize this essential host pathway could prove a viable avenue for improving mycobacterial vaccines (Yuk and Jo, 2014; Flores-Valdez et al., 2018; Tao and Drexler, 2020). Notably, the yellow fever vaccine, YF-17D, one of the most successful vaccines, has been found to enhance autophagy-dependent antigen presentation. The mechanisms of the vaccine efficacy by YF-17D were not well understood until its role in autophagy modulation was deciphered (Ravindran et al., 2014).

Developing new vaccines or improving the BCG by harnessing autophagy is an area of interest and has garnered examination. BCG, like *Mtb*, expresses a wide array of bacterial effectors that modulate autophagy, but co-immunization of mice with BCG and rapamycin-treated dendritic cells enhanced Th1-mediated protection against *Mtb* infection (Jagannath et al., 2009). Similarly, it was demonstrated that a recombinant BCG expressing 85C5 (BCG^85*C*5^) induced a robust MHC-II-dependent antigen presentation to CD4^+^ T cells *in vitro*. The 85C5 peptide contains the TLR-2 activating C5 peptide from *Mtb* CFP-10 protein. The vaccine also elicited stronger Th1 cytokines from APCs of C57Bl/6 mice and enhanced MHC-II surface expression on macrophages by inhibiting the membrane associated RING-CH 1 (MARCH1) E3 ligase that degrades MHC-II. BCG^85*C*5^ infected APCs presented antigens in a MyD88 or a TLR-2 dependent manner (Khan et al., 2019). Additionally, activation of TLR3 or TLR4 by LPS cleared mycobacteria *in vitro* in an autophagy-dependent way (Xu et al., 2007, 2013).

BCG was genetically modified to improve its immunogenicity by replacing the urease C encoding gene with the listeriolysin encoding gene from *Listeria monocytogenes* (Nieuwenhuizen et al., 2017). Listeriolysin perturbates the phagosomal membrane at acidic pH and Urease C neutralize the phagosome harboring BCG. Deletion of *ureC* leads to rapid phagosome acidification and promotes phagolysosome fusion. Subsequently, BCGΔureC:hly elevates apoptosis and autophagy and accelerates release of mycobacterial antigens into the cytosol. The BCGΔ*ureC*:*hly* vaccine completed phase I and IIa clinical trials. Upon deleting the anti-apoptotic gene *nuoG* to enhance cross protection, BCGΔ*ureC*:*hly*Δ*nuoG* vaccine showed reduced *Mtb* burden in the lungs of mice leading to less pathology and, most importantly, enhanced immune responses. It was found that the *nuoG* deletion leads to significant induction of autophagy and an improved safety profile (Gengenbacher et al., 2016). *M. indicus pranii* is another potential immunotherapy and vaccine candidate under clinical trials (Gupta et al., 2012; Saqib et al., 2016; Sharma et al., 2017). Boosting BCG vaccination with *M. indicus pranii* resulted in improved protection in a murine model of *Mtb* infection. Increased IFN-γ, IL-12, and IL-17 were observed along with increased polyfunctional T cells (Saqib et al., 2016). This increased protection and immune response were subsequently due to increased autophagy induced by *M. indicus pranii*, potentially due to its PILAM (Singh et al., 2017, 2019).

While improving the BCG vaccine appears a viable short-term solution to improve vaccine efficacy against *Mtb*, the efforts to develop new vaccines must be continued. BCG is a live attenuated vaccine, but it is not suitable for use in all cases. The development of other vaccine options, such as DNA vaccines or subunit vaccines, would significantly advance the vaccination strategy against *Mtb* and NTMs. A DNA vaccine encoding for the potent *Mtb* antigen 85B (Ag85B) significantly enhanced autophagy activation and vaccine efficacy when delivered with a plasmid encoding a kinase defective (mTOR-KD) (Meerak et al., 2013). This mTOR-KD DNA vaccine-elicited considerably higher Ag85B-specific antibodies, increased secretion of IFN-γ and IL-2 levels, and enhanced proliferation of CD4^+^ T cells. Similar to this approach, it was found that an LC3-LpqH-Ag85B DNA vaccination reduced mycobacterial burden, increased IFN-γ and IL-2, and enhanced the Th1 immune response (Hu et al., 2014).

Adjuvating or boosting BCG with autophagy-inducing substrates has also been examined as a potential way to increase the efficacy of current vaccines. Curcumin-coated nanoparticles have been found to enhance autophagy, leading to increased Th1 and Th17 central memory T cells (Ahmad et al., 2019). Other studies have identified autophagy as having an essential role in forming and surviving memory T cells (Xu et al., 2014). Like curcumin nanoparticles, boosting BCG vaccine with nanofibers acting through the autophagy pathway improved BCG efficacy (Rudra et al., 2017; Chesson et al., 2018). While some DNA vaccines directly target autophagy, the development of an adjuvant system targeting autophagy may improve the efficacy of potential subunit vaccines. Utilizing the lactic acid bacteria (LAB) as an adjuvant for *Mtb* antigens showed improved IFN-γ and NO responses, polarizing a Th1 response and increasing autophagosome formation. Although LAB’s protective efficacy was not tested, these improved immunological responses compared to *Mtb* antigen alone are promising (Ghadimi et al., 2010).

## Conclusion

Autophagy has been established as an effective mechanism for the clearance of mycobacteria from the infected macrophages. Many studies have looked at the potential of autophagy-inducing drugs to improve current treatment regimens against mycobacteria. Due to the dramatic rise of antibiotic-resistant mycobacteria and the upsurge of NTMs that are intrinsically resistant to traditional antibiotics, host-directed therapies are even more relevant (Deretic, 2008; Kim et al., 2012; Zullo and Lee, 2012b; Kim et al., 2019; Bento et al., 2020).

While *Mtb* inhibits apoptosis for bacterial survival (Hinchey et al., 2007), NTMs utilize the hosts’ cellular progression from apoptosis to secondary necrosis (Gao and Kwaik, 2000; Lee et al., 2011) or induce membrane perforation (similar to that observed during necrosis) to allow for bacterial escape and communication (Roux et al., 2016). Subsequent expression of bacterial factors that form an extracellular milieu or biofilm makes control by either phagocytic cells or administered antibiotics much more difficult. If host-directed therapies inhibiting apoptosis or inducing autophagy could be employed against these non-tuberculosis mycobacteria, infection control of the contained intracellular mycobacteria with traditional antibiotics may be far more successful. Additionally, it has been demonstrated that potent autophagy-inducing chemicals could increase mycobacterial clearance from macrophages, like seen during rapamycin treatment (Singh et al., 2017).

Autophagy-targeting host-directed therapies and vaccines for mycobacteria have numerous potential benefits. However, further understanding of the role of autophagy, its molecular mechanisms, and regulation during mycobacterial infection is required to develop persistent, viable, and safe host-directed therapies and vaccines. Additionally, examination of the crosstalk between autophagy and apoptosis during infection should significantly improve our understanding of the applicability of these host pathways as a viable target for treatment. Though host-directed therapies may play a vital role for intrinsically antibiotic-resistant NTMs and drug-resistant TB, they will need to be considered together with traditional anti-mycobacterial medicines, with the goal of shorter treatment times and improved outcomes.

## Author Contributions

ES and SL conceived the review. ES wrote the first draft of the manuscript. SL wrote sections of the manuscript and provided overall editing. All authors contributed to manuscript revision, read, and approved the submitted version.

## Conflict of Interest

The authors declare that the research was conducted in the absence of any commercial or financial relationships that could be construed as a potential conflict of interest.
